# Adrenergic receptor activation triggers stress-induced dystonia in a CACNA1A mutant mouse model

**DOI:** 10.3389/fnins.2026.1765171

**Published:** 2026-03-02

**Authors:** Pauline Bohne, Michelle Grömmke, Max Rybarski, Tejas Nair, Melanie D. Mark

**Affiliations:** 1 Department of Behavioral Neuroscience, Ruhr-University Bochum, Bochum, Germany; 2 Department of Zoology and Neurobiology, Ruhr-University Bochum, Bochum, Germany

**Keywords:** adrenergic receptors, cerebellum, dystonia, episodic ataxia type 2, stress

## Abstract

Episodic ataxia type 2 (EA2) is caused by loss-of-function mutations in CACNA1A, resulting in P/Q-type Ca^2+^ channel dysfunction in cerebellar Purkinje cells (PCs) causing ataxia and stress-induced dystonia. Using Cacna1a^purk(−/−)^ (*purky*) mice, which display selective P/Q-type channels deletion in PCs, the effects of adrenergic receptor (AR) blockade on stress-induced dystonia were examined. Systemic administration of the α1-AR antagonist prazosin increased dystonia frequency, but shortened attack duration, while the α1D-AR selective antagonist BMY-7378 significantly reduced dystonia occurrence without altering onset or duration. Strikingly, universal blockade of α2-ARs using yohimbine, as well as agonist of α2A-AR autoreceptors completely abolished stress-induced dystonia. Electrophysiological recordings of cerebellar PCs demonstrated that norepinephrine (NE) strongly inhibited the PC simple spike firing, which was partially rescued by yohimbine, implicating α2-AR–dependent modulation of PC activity. Histological analysis of *purky* mice revealed increased dopamine-*β*-hydroxylase (DβH) immunoreactivity on PC somata, which was accompanied by increased numbers of noradrenergic neurons in locus coeruleus (LC), indicating enhanced cerebellar noradrenergic innervation. These findings strengthen the idea that stress-induced dystonia formation is facilitated by increased noradrenergic innervation to cerebellar PCs and suggest that α2-AR signalling contributes to dystonia in EA2. Our findings emphasise cerebellar ARs as promising therapeutic targets in EA2.

## Introduction

Episodic ataxia type 2 (EA2) is a rare autosomal dominant inherited neurological disorder caused by loss-of-function mutations in the CACNA1A gene, which encodes the pore-forming α1A-subunit of the voltage-gated P/Q-type calcium channel (Ophoff et al., 1996). Dysfunctions in the channel lead to decreased Ca^2+^ influx in cerebellar Purkinje cells (PCs), the sole output neuron of the cerebellar cortex. Clinically, EA2 is characterized by recurrent episodes of ataxia, vertigo and dystonia which can last a few hours to days and can be triggered by physical or emotional stress, ethanol or caffeine (Jen and Wan, 2018).

To elucidate the pathophysiology of EA2, several mouse models have been developed (Mark et al., 2011; Maejima et al., 2013; Fureman and Hess, 2005). The tottering^tg/tg^ mouse, a well-established mouse model for EA2, displays a spontaneous mutation in the *Cacna1a* gene, resulting in ataxia and stress-induced dystonia associated with aberrant PC activity (Walter et al., 2006). Recent work from our laboratory demonstrated that selective blockade of α1D-adrenergic receptors (ARs) is sufficient to abolish stress-induced dystonia and improve ataxia in tottering^tg/tg^ mice by stabilizing PC firing (Bohne et al., 2025), suggesting α1-AR blockade as a potential treatment option (Snell et al., 2022). Our laboratory previously established Cacna1a^purk(-/-)^ mice (*purky*) as a complementary EA2 model. In purky mice, the P/Q-type calcium channel is selectively deleted from PCs, resulting in severe ataxia and paroxysmal stress-induced dystonia (Mark et al., 2011), providing a valuable system for dissecting PC-dependent mechanisms in EA2 pathophysiology. In this study we aimed to identify noradrenergic receptor subtypes mediating stress induced dystonia in *purky* mice, where the P/Q-type calcium channel is specifically ablated from PCs.

## Materials and methods

### Animals and genotyping

Data were obtained in adult Cacna1a^Citrine^ (control) and Cacna1a^purk(−/−)^ mice (24–52 weeks). Cacna1a^Citrine^ mice (Mark et al., 2011) were bred with Tg^pcp2-Cre^ mice (JAX stock #004146; B6.129-Tg(Pcp2-cre)2Mpin/J) (Barski et al., 2000) to obtain homozygous Cacna1a^purk(−/−)^. Genomic tail biopsies were performed to verify the genetic background of mice. Using the following primers: *Cacna1a forward* 5′ GGGGTCTGACTTCTGATGGA 3′, *reverse* 5′ AAGTTGCACACAGGGCTTCT 3′; *Cacna1a^Citrine^ forward* 5′ TATATCATGGCCGACAAGCA 3′, *reverse* 5′ TTCGGTCTTCACAAGGAACC 3′, *Tg^pcp2 − Cre^ forward* 5′ ATTCTC CCACCACCGTCAGTACG 3′, *reverse* 5′ AAAATTTGCCTGCATTACCG 3′. Mice were single and group housed on a 12 h light/dark cycle and *ad libitum* access to food and water. All experiments were conducted in accordance with the European Communities Council Directive of 2010 (2010/63/EU) for care of laboratory animals and approved by the local ethics committee (Bezirksamt Arnsberg) and the animal care committee of North Rhine-Westphalia, Germany, based at the LAVE (Landesamt für Verbraucherschutz und Ernährung, Nordrhein-Westfalen, D-45659 Recklinghausen, Germany). The study was supervised by the animal welfare commission of the Ruhr-University Bochum.

### Stress-induced dystonia

Stress-induced dystonia was triggered by exposing the animal to a fresh cage individually (cage change stress) as previously described (Bohne et al., 2025). Briefly, mice were observed for the occurrence of dystonic episodes for 40 min. In the absence of dystonia, mice were removed to their familiar home cage. The onset and duration were noted in dystonic animals. The frequency [%] of dystonia reflects the percentage of mice experiencing a stress-induced episode in presence of the tested drug of all mice tested. After the episode ceased, animals were returned to their home cages. Mice had permanent access to food and water. There was at least one recovery day between cage change stress tests.

### Drug administration

Stock solutions of prazosin hydrochloride (Sigma Aldrich, P7791), yohimbine hydrochloride (Sigma Aldrich, Y3125), clonidine hydrochloride (Sigma-Aldrich, C7897) and BMY-7378 dihydrochloride (Tocris, #1006) were prepared in distilled water and stored as recommended by the manufacturer. Working solutions were prepared in sterile NaCl in doses of 1, 2.5, 5 and 10 mg/kg for prazosin, 10 mg/kg for BMY-7378, 1, 2.5, 5, 10 and 20 mg/kg for yohimbine and 0.0125, 0.025, 0.05 and 0.1 mg/kg for clonidine. Mice were intraperitoneally injected 30 min prior to the cage change stress test. Different groups of mice were used for each tested drug.

### Motor experiments

To assess the potential positive effects of *α*2-adrenergic receptor blocker yohimbine on ataxia and motor coordination, the motor tests beam walk, gait analysis, hang wire, pole test and rotarod were performed according to previous methods (Mark et al., 2011; Maejima et al., 2013). Cacna1a^Citrine^ and Cacna1a^purk(−/−)^ mice were injected with either vehicle (NaCl) or yohimbine (20 mg/kg) 30 min prior to the motor test. One test/day was performed and mice were given at least 1 day to recover.

### Histology

4–8 months old mice were deeply anesthetized with ketamine/xylazine (150/20 mg/kg, respectively) and transcardially perfused with 1× PBS followed by ice-cold 4% FA in 1× PBS (pH 7.4, Sigma-Aldrich) as previously described (Bohne et al., 2025). Brains were dissected and post-fixed for 1 h in 4% FA, then cryoprotected in 30% sucrose in PBS overnight before slicing on a Leica CM3050 S cryostat.

#### Dopamine-*β*-hydroxylase/calbindin staining

Noradrenergic contacts onto cerebellar PCs were visualized by co-staining of dopamine-β-hydroxylase (Invitrogen, PA5-34664) and calbindin (Sigma Aldrich, C9848). 35 μM coronal cerebellar sections were blocked in 3% NDS in 0.2% PBS-T for 60 min before incubation with rabbit α-DβH (1,1,000) and mouse α-Calbindin (1,500) in blocking medium overnight at 4 °C. Sections were washed three times in 1x PBS followed by secondary antibody incubation of goat α-rabbit DyLight 649 (Invitrogen, SA5-10029) and donkey α-mouse Alexa Fluor™ 488 (Invitrogen, A-21202) (each 1:1000) in blocking solution for 3 h. The slices were mounted using ROTI® mount FluorCare Dapi (Carl ROTH) and stored at 4 °C until imaging.

#### Locus coeruleus analysis

Noradrenergic neurons in the locus coeruleus (LC) were visualized by dopamine-β-hydroxylase staining. 35 μm thick slices of the LC were prepared and stained as stated above. Dopamine-β-hydroxylase positive neurons were counted manually. To determine the LC area, we manually encircled the DβH^+^ area of the LC using ImageJ. Within this area, DβH^+^ pixel were analyzed using the local threshold algorithm Phansalkar with a rolling ball radius of 30 μm.

### Imaging

All images were obtained using an inverted Leica TCS SP5 confocal laser scanning microscope (Leica DMI6000 B, Wetzlar Germany) interfaced to a computer running the Leica Application Suite Advanced Fluorescence software (LAS AF 2.6). Sequential z-stacks of 10 to 15 images were performed for each section. Acquired images were analyzed using ImageJ (NIH).

#### Dopamine-β-hydroxylase (DβH) intensity analysis

To quantitatively compare the noradrenergic innervation to Purkinje cells of control and mutant Cacna1a^purk(−/−)^ mice, three images at 40x from each lobule 4 and 5, lobule 9 and Crus 2 were taken from cerebellar slices stained with calbindin and DβH. Images were analyzed for the soma area and mean fluorescence intensity (mFl) of DβH staining in PC soma by manually encircling the calbindin positive soma using ImageJ. Identical imaging conditions were used for all mice analyzed. The ratio was formed by dividing the mFl by the PC soma area.

### Electrophysiology

#### Recordings

Electrophysiological recordings were conducted as previously described (Bohne et al., 2025). For microinjection of adrenoreceptor modulators diluted in ACSF, quartz glass microinjection pipettes (outer diameter: 115 μm, inner diameter: 85 μm; Thomas Recording, Giessen, Germany) were positioned next to the recorded cell. 10 mM norepinephrine (Sigma Aldrich, A7257) was applied after 60 s of reference recording via pressure injection. NE effects were recorded for 70 s, followed by application of 5 μM yohimbine hydrochloride (Sigma Aldrich, Y3125). Recordings were conducted for a total duration of 300 s and saved for offline analysis.

#### Data analysis

Offline analysis of recorded traces was conducted as previously described using a custom-made software implemented in Matlab (Bohne et al., 2025).

### Statistical analysis

Test procedure, statistical significances and number of animals (*N*) or cells analyzed (*n*) for each experiment can be found in the Supplementary tables. Statistical analysis was conducted with SigmaPlot (Systat Software), the level of significance was set to *p* ≤ 0.05. Error bars display mean ± SEM, if not stated otherwise Statistical significance is reported as n.s (not significant); *p ≤ 0.05; **p ≤ 0.01; ***p ≤ 0.001.

## Results and discussion

**Figure 1:**
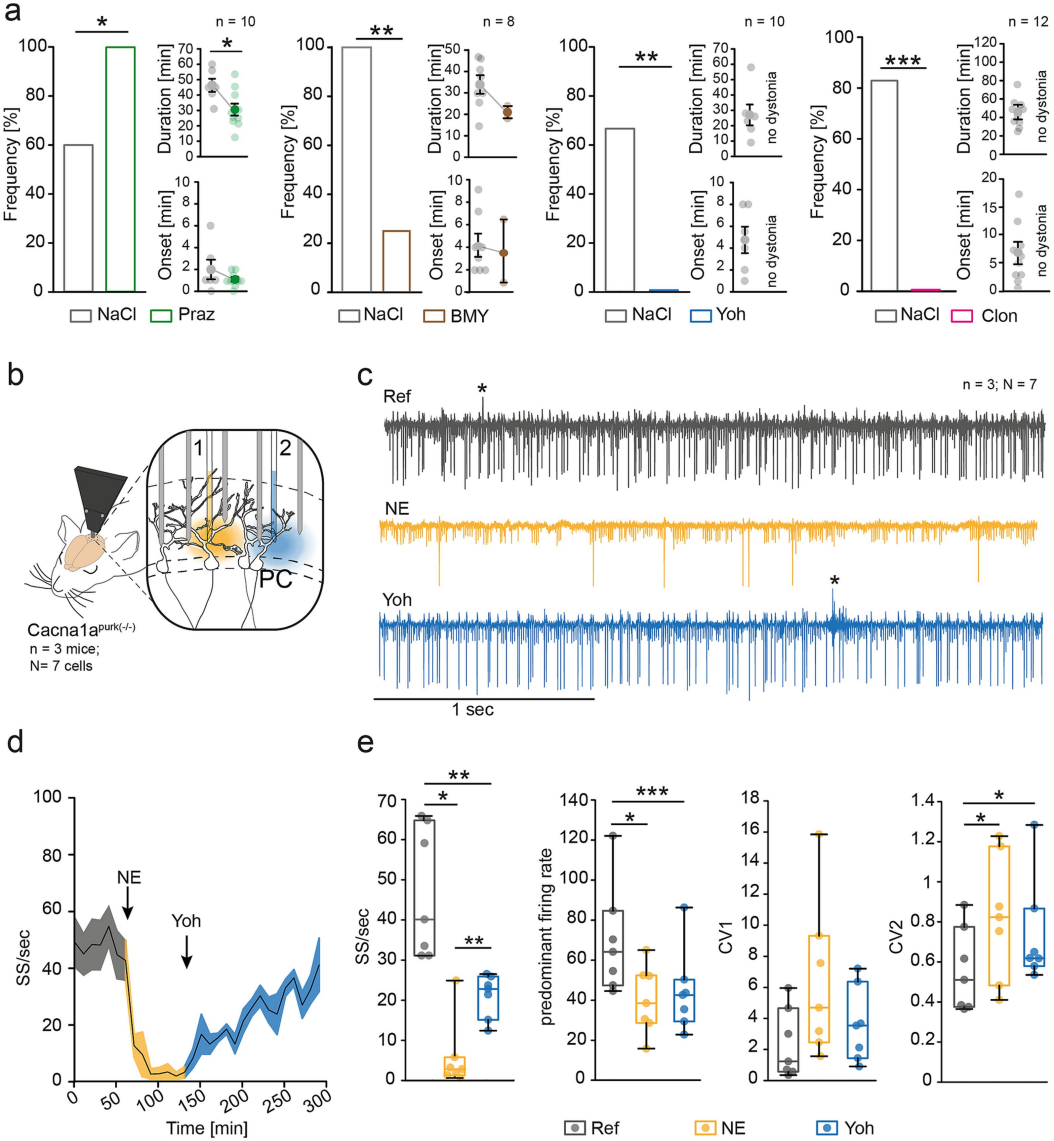
α-Adrenergic receptor blockade alleviates stress-induced dystonia by restoring Purkinje cell firing in *Cacna1a*^*purk*(−/−)^ mice. **(a)** Pharmacological antagonism of α1-ARs using the general α1-AR antagonist prazosin (praz, green, 10 mg/kg) exacerbated stress-induced dystonia compared to vehicle injected mice (grey, *n* = 10, *p* = 0.034) but decreased the duration of attacks after the cage change stress. Blockade of specifically α1D-ARs using 10 mg/kg BMY-7378 (brown, *n* = 8) significantly reduced stress-induced dystonia in *Cacna1a*^*purk*(−/−)^ mice (*p* = 0.01). The blockade of general α2-ARs using 20 mg/kg yohimbine (yoh, blue, *n* = 10), but also specifically agonizes presynaptic α2A adrenergic autoreceptors using 0.1 mg/kg clonidine (pink, *n* = 12) alleviated stress-induced dystonia completely compared to vehicle injected mice. **(b)** Scheme of extracellular PC recordings in anaesthetized *Cacna1a*^*purk*(−/−)^ mice (*n* = 3 mice, *N* = 7 cells) using a multielectrode manipulator. To mimic the endogenous release of norepinephrine (NE), 10 mM NE (yellow) was pressure-injected into the cerebellar cortex, followed by pressure-injection of 5 μM Yoh (blue). **(c)** Example raw traces of PC simple spikes (SS) from *Cacna1a*^*purk*(−/−)^ mice under reference conditions (Ref, grey), after NE (yellow) and Yoh (blue). Complex spikes are indicated with star (*). **(d)** Mean trace of PC firing alterations shows that the NE-induced inhibition of PC SS firing was partially restored after application of Yoh. **(e)** Norepinephrine significantly reduced the firing frequency of recorded PCs by 87% (*p* = 0.016) and increased the intrinsic PC irregularity by 47.13% (CV2, *p* = 0.029). Subsequent application of Yoh partially recovers PC SS firing from 5.8 ± 3.245 to 21.114 ± 2.033 (*p* = 0.004).

To investigate whether the beneficial effects of AR blockade observed in tottering^tg/tg^ mice can also be transferred to *purky* mice, we intraperitoneally injected different α1-, and α2-AR antagonists and exposed the animals to a new environment (cage change stress) (Figure 1). The general α1-AR prazosin (Praz, green, 10 mg/kg) increased the frequency of cage change stress-induced dystonia in Cacna1a^purk(−/−)^ mice from 60 to 100% (*n* = 10), but reduced dystonia duration compared to vehicle injected *purky* mice (Figure 1a). Interestingly, the α1D-AR subtype specific antagonist BMY-7378 (brown, 10 mg/kg) substantially alleviated stress-induced dystonia by 75%, without affecting duration or onset of attacks (*n* = 8). A complete absence of stress-induced dystonia was observed when we blocked α2-ARs (Figure 1a) with both the general α2-AR blocker yohimbine (Yoh, blue, *n* = 10), as well as the α2A adrenergic autoreceptor agonist clonidine (Clon, pink, *n* = 12) (Figure 1a). Clonidine primarily binds to presynaptically expressed α2-AR, thereby decreasing the release of NE and abolishing stress-induced dystonia in a dose-dependent manner (Fureman and Hess, 2005). Yohimbine, however, blocks α2-ARs, therefore likely preventing NE-induced effects postsynaptically. However, injection of Yoh had no rescuing effect on ataxia in purky mice (Table 1) and did not improve motor coordination in control mice (Supplementary Table 3). Interestingly, tottering^tg/tg^ mice do not show alleviation of stress-induced dystonia after yoh injection (Fureman and Hess, 2005; Bohne et al., 2025), thus our *purky* mice are the first EA2 model which respond to α2-, and α1D-AR blockade. Since yohimbine was effectively preventing stress-induced dystonia in *purky* mice, we explored the effects of α2-AR blockade using Yoh on the norepinephrine (NE)-induced inhibition of PC simple spike (SS) firing in anaesthetized Cacna1a^purk(−/−)^ mice (Figure 1b, *N* = 3 mice). Pressure injection of 10 μM NE inhibited PC SS firing by 87% compared to baseline recordings (Ref, grey, *N* = 7 cells) (Figures 1c–e). However, application of 5 μM Yoh significantly recovered the PC SS firing, suggesting a rescinding effect on the NE-induced inhibition (Figure 1e). Surprisingly, we did not find positive effects of Yoh on PC regularity or the predominant firing rate of PCs, suggesting contributions of other cerebellar neurons to alleviation of dystonia by α2-AR blockade in *purky* mice.

**Table 1:** Yohimbine hydrochloride does not improve ataxia in Cacna1a^purk(−/−)^ mice (*n* = 11).

Test	Statistics	mean ± SEM		*p*-value
		NaCl	Yohimbine	
Pole test time (s)	Mann–Whitney rank sum test	115.591 ± 4.409	120.0 ± 0.0	*p* = 0.363
Hang wire time (s)	Mann–Whitney rank sum test	7.182 ± 1.979	2.848 ± 0.464	*p* = 0.061
Beam walk				
Time (s)	Mann–Whitney rank sum test	120.0 ± 0.0	120.0 ± 0.0	*p* = 1
Idle (s)	–	–	–	–
Falls (*n*)	Mann–Whitney rank sum test	11	11	p = 1
Right slips (*n*)	–	–	–	–
Left slips (*n*)	–	–	–	–
Footprint analysis				
Length right front paw (cm)	Students *T*-tests	4.852 ± 0.232	4.299 ± 0.134	*p* = 0.054
Length left front paw (cm)	Students *T*-tests	4.723 ± 0.773	4.210 ± 0.171	*p* = 0.103
Length right hind paw (cm)	Students *T*-tests	4.693 ± 0.193	4.427 ± 0.182	*p* = 0.332
Length left hind paw (cm)	Students *T*-tests	4.787 ± 0.229	4.239 ± 0.177	*p* = 0.075
Width front paws (cm)	Students *T*-tests	2.135 ± 0.103	1.930 ± 0.105	*p* = 0.181
Width hind paws (cm)	Mann–Whitney rank sum test	2.743 ± 0.170	2.834 ± 0.214	*p* = 0.940

Cacna1a^putk(−/−)^ could not perform the beam walk test and did not leave the platform. If placed on the beam directly, mice would fall.

**Figure 2:**
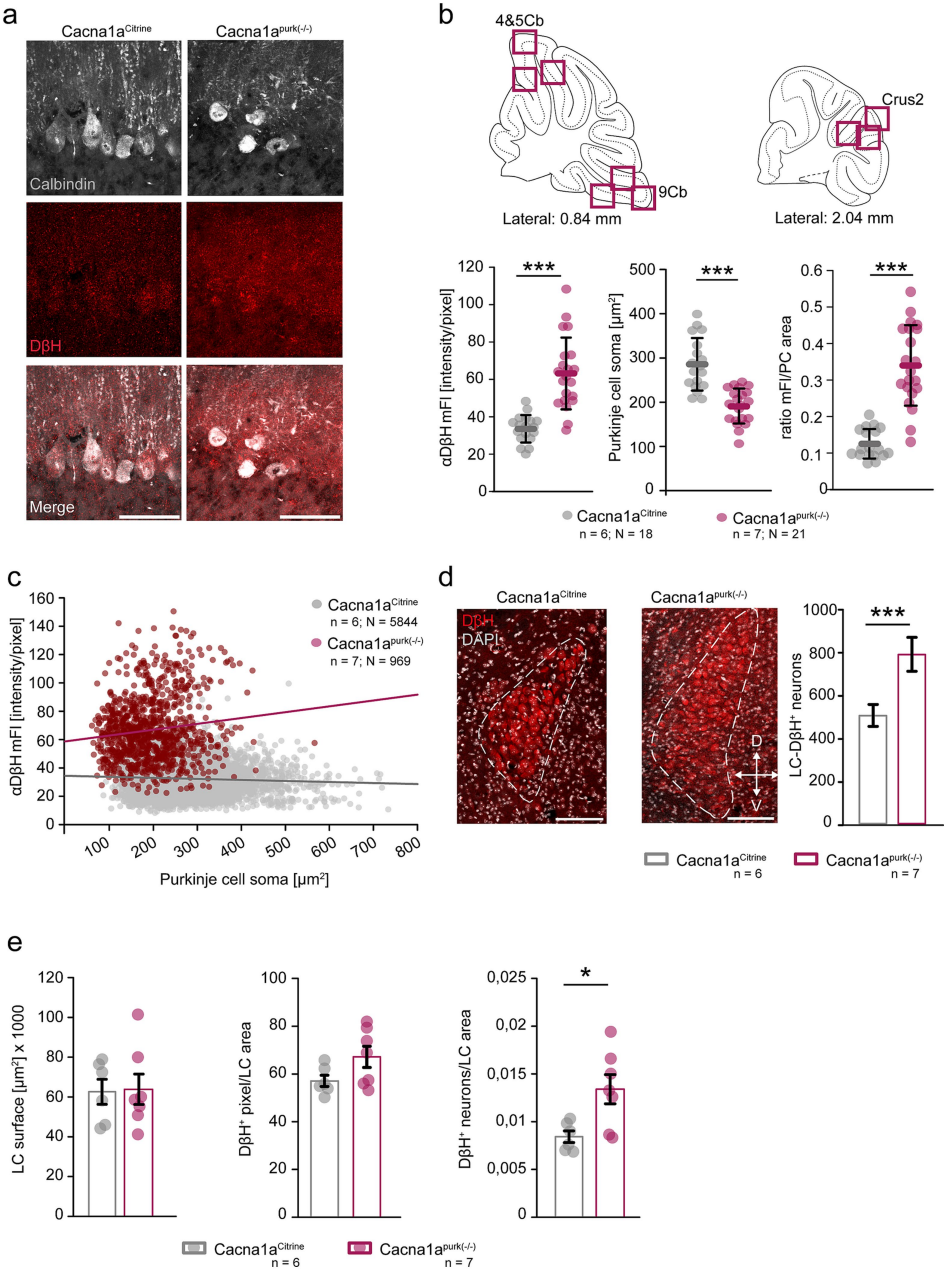
*Cacna1a*^*purk*(−/−)^ mice display PC degeneration and increased noradrenergic innervation. **(a)** Representative confocal images of cerebellar coronal sections depicting PCs identified by calbindin staining (grey) and the immunoreactivity to DβH (red) in the molecular layer and PC soma (merge, white) in *Cacna1a^Citrine^* (control) and *Cacna1a*^*purk*(−/−)^ mice. Scale bars: 50 μm. **(b)** Three 40 x images per cerebellar lobule 4 and 5, lobule 9 and Crus 2 (magenta squares) were taken from control (grey) and *Cacna1a*^*purk*(−/−)^ mice (magenta) to analyze Purkinje cell somata for their immunoreactivity (shown as mean fluorescence (mFl) intensity) to DβH and their soma size. The mFl of PC soma was significantly higher in *Cacna1a*^*purk*(−/−)^ mice compared to control mice (Mann–Whitney *U*-test, *p* ≤ 0.001), while their PC soma were significantly smaller (*t*-test, *p* ≤ 0.001) and the ratio of the mFl to PC soma area was higher in *Cacna1a*^*purk*(−/−)^ mice (Mann–Whitney *U*-test, *p* ≤ 0.001). **(c)** The mFl of DβH staining was plotted against PC soma size of all PCs analyzed in both *Cacna1a^Citrine^* control and *Cacna1a*^*purk*(−/−)^ mice. Purkinje cell soma in *Cacna1a*^*purk*(−/−)^ mice, although smaller, showed stronger DβH fluorescence intensities, suggesting increased noradrenergic innervation. **(d)** Analysis of the LC (dotted line) revealed significantly more DβH^+^ neurons in *Cacna1a*^*purk*(−/−)^ (magenta) compared to control *Cacnca1a^Citrine^* mice (grey) (*t*-test, *p* = 0.002). Scale bar: 100 μm. **(e)** Analysis of the LC surface area (*p* = 0.908), as well as the amount of DβH^+^ pixels within the LC areas did not show any differences (*p* = 0.084) between *Cacnca1a^Citrine^* and *Cacna1a*^*purk*(−/−)^ mice. However, we found that *Cacna1a*^*purk*(−/−)^ mice had significantly more DβH^+*^ neurons in the same LC surface area, suggesting that although the LC itself is not enlarged, *purky* mice display altered noradrenergic LC neuron physiology. All data are presented as mean ± SEM.

Norepinephrine containing terminals in the cerebellum originate solely from the locus coeruleus (LC) (Olson and Fuxe, 1971). The LC-NE^+^ fibres mainly form synapses with PC dendrites (Olson and Fuxe, 1971; Bloom et al., 1971; Hökfelt and Fuxe, 1969), but also synapse on PC soma, granule cell dendrites, and the deep cerebellar nuclei (Gould et al., 1997). Interestingly, several cerebellar degeneration animal models show alterations in their cerebellar monoaminergic system (Landis et al., 1975; Muramoto et al., 1982). For example, tottering^tg/tg^ mice display increased noradrenergic innervation (Levitt and Noebels, 1981), while the number of LC neurons is not altered (Noebels, 1984). Similarly, P*urkinje cell degeneration* (*pcd*) mice, a mouse model of human degenerative ataxia, display PC loss accompanied by cerebellar degradation and ataxia (Mullen et al., 1976), similar to what we observe in our *purky* mice (Mark et al., 2011). The *pcd* mouse exhibits increased amounts of NE transporters in the cerebellar cortex, especially in vermis and paravermis which are associated with motor functions. To investigate whether dystonia is accompanied by altered noradrenergic innervation in our Cacna1a^purk(−/−)^ mice, we performed immunohistochemical staining against dopamine-*β*-hydroxylase (DβH, red), the enzyme which catalyses dopamine to norepinephrine, in motor coordination involved cerebellar lobules 4 and 5, vestibular controlling lobule 9 and cognition related Crus2 (Figure 2). In accordance with the literature, we found severe loss of PCs in *purky* mice in all regions analysed (Mark et al., 2011; Ghetti et al., 1981; Todorov et al., 2012). Remaining PCs were identified by calbindin staining (white, Figure 2a), which revealed significantly smaller somata compared to control mice (Figure 2b). However, *purky* PCs showed increased DβH immunoreactivity, displayed as mean fluorescence intensities (mFl), compared to control mice (Figure 2b). This also reflects in a higher ratio of the mFl to PC area in *purky* meaning smaller PC soma exhibit brighter fluorescence, suggesting increased noradrenergic innervation from the LC to PCs (Figure 2c). In addition, we also found more DβH^+^ soma in the LC of *purky* compared to control mice, potentially suggesting that the increased DβH^+^ immunoreactivity in the cerebella of *purky* mice is a consequence of an increased number of neurons in the LC projecting to the PC layer (Figure 2d). To confirm this hypothesis, we additionally analysed the area comprising the LC in both control and Cacna1a^purk(−/−)^ mice. We could not identify alterations in the LC size or the amount of DβH^+^ pixels within this area, but analysis revealed that *purky* mice display increased neuronal density in the LC, suggesting alterations in neurogenesis (Figure 2e). Our findings strengthen the idea that EA2 and the formation of stress-induced dystonia is correlated to altered noradrenergic innervation.

Many studies correlated AR activation to stress-induced dystonia in EA2 mouse models (Fureman and Hess, 2005; Bohne et al., 2025; Snell et al., 2022; Fureman et al., 2002). A predominant role of α1-ARs is commonly accepted, and we recently reported the contribution of α1D-AR specifically expressed on PCs to dystonia formation in tottering^tg/tg^ mice (Bohne et al., 2025). Interestingly, both the specific α1D-AR blocker BMY-7378, but also general α2-AR yohimbine alleviated stress-induced dystonia in *purky* mice, suggesting a coordinated engagement of α1- and α2-ARs during dystonia formation. In mice, α1D-ARs are almost exclusively expressed in cerebellar PCs^7^ and both the pharmacological block, as well as shRNA-induced knock-down of specifically cerebellar α1D-ARs alleviated stress-induced dystonia in tottering^tg/tg^ mice (Bohne et al., 2025), suggesting a PC specific effect. Similarly, blocking α1D-ARs in *purky* mice decreased, but not abolished, stress-induced dystonia, suggesting that both α1D-ARs and PCs are required for dystonia in *purky* mice, but are likely not the only key players. Injection of yoh alleviated stress-induced dystonia, possibly by simultaneously blocking multiple α2-AR subtypes expressed throughout the cerebellar network (Bohne et al., 2025; Wang et al., 1996). This interpretation is based on previously published studies linking dystonia to the cerebellum (Bohne et al., 2025; Snell et al., 2022). Since yoh was administered systemically, we cannot exclude contributions from other brain regions or peripheral off targets. Our hypothesis of yoh targeting multiple subtypes simultaneously and thereby preventing dystonia, is strengthened by the selective blockade of α2B-ARs via i.p. injection of imiloxan hydrochloride which did not alleviate stress-induced dystonia in *purky* mice (Supplementary Figure 1). This observation suggests that under our experimental conditions, selective blockade of α2B-ARs is insufficient to modify dystonia and that the other α2 subtypes or additional mechanisms are involved.

We hypothesise that yohimbine antagonizes different subtypes of α2-ARs in the cerebellar network, thereby attenuating the NE-induced inhibition of PCs, as demonstrated in our electrophysiological recordings (Figure 1). However, our electrophysiological recordings show only a partial restoration, which may not fully explain the observed phenotypical effect of complete stress-induced dystonia abolishment. A likely explanation could be that NE already activated both α1- and α2-ARs and respective G-protein pathways, where belated injection of yoh only partially reversed these NE-induced effects. We previously made these observations for NE-induced inhibition of PCs in tottering^tg/tg^ mice using BMY-7378, where BMY-7378 showed better protection of PC SS firing when injected before NE (Bohne et al., 2025). Similar effects could apply to yoh, too. Additionally, we recorded from anaesthetized mice, thus the observed effects do not necessarily correlate to the observed behavioural effects. Lastly, since we only recorded from PCs, we cannot rule out the effects of other cerebellar neurons on PCs or the whole cerebellar network, which could contribute to the absence of dystonia. In accordance with this hypothesis, a recent study showed that activation of presynaptic α2A- and α2B-ARs downregulated parallel fibre (PF) to PC synaptic transmission (Wang et al., 2023), thereby reducing glutamatergic-induced SS firing. The PCs of *purky* mice display severe firing deficits (Mark et al., 2011), but in the presence of NE, the firing can be further decreased via presynaptic activation of α2A- and α2B-ARs on the PF synapse. Blocking the NE-mediated activation of presynaptic α2A- and α2B-ARs using yohimbine could promote normal PC excitation and help maintain physiological PC firing.

Our data suggest that stress-induced dystonia in Cacna1a^purk(−/−)^ mice involves both α1D- and α2-adrenergic receptor signalling, thereby strengthening ARs as therapeutic targets in EA2. However, given that our conclusions are based on a specific genetic mouse model, systemic pharmacological manipulations and a particular stress paradigm, further studies using more selective and region-specific interventions, different EA2 models and ideally human data will be required to fully assess the translational potential and safety of targeting adrenergic receptors in EA2.

## Data Availability

The original contributions presented in the study are included in the article/Supplementary material, further inquiries can be directed to the corresponding author.
