# Supplementary material for: Adrenergic receptor activation triggers stress-induced dystonia in a CACNA1A mutant mouse model
Source: Front Neurosci. 2026 Mar 2;20:1765171. doi: 10.3389/fnins.2026.1765171 (PMC12989501; doi:10.3389/fnins.2026.1765171)
Supplement: Supplementary file 1 [file Data_Sheet_1.docx]

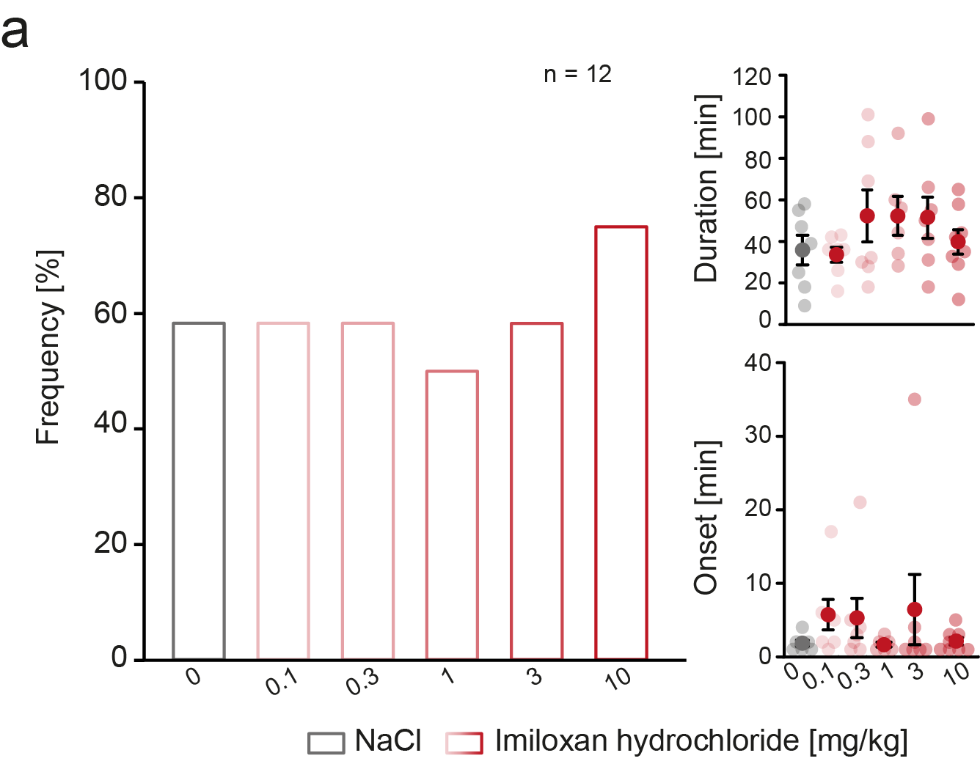


Supplementary Figure 1: Blocking of α2B-AR using Imiloxan hydrochloride has no influence on the frequency, duration or onset of stress-induced dystonia in Cacna1a^purk(-/-)^ mice. Different concentrations were i.p. injected, followed by a cage change stress-test in Cacna1a^purk(-/-)^ mice.

Supplementary Table 1: Mean±SEM, statistical tests and p-values for main data.

| **Figure 1** |  | **mean±SEM** | | | | **Statistical test** | **p-value** |
| --- | --- | --- | --- | --- | --- | --- | --- |
|  | **parameter** | NaCl  N=10 | | Praz  N=10 | |  |  |
| **a** | frequency | 60%±0% | | 100%±0% | | Mann-Whitney U-test | **p=0.034** |
|  | duration | 46.333±4.271 | | 30.55±3.894 | | t-test | **p=0.02** |
|  | onset | 2.0±0.894 | | 1.1±0.615 | |  | p=0.685 |
|  |  | NaCl  N=8 | | BMY-7378  N=8 | |  |  |
|  | frequency | 100%±0% | | 25%±0% | | Mann-Whitney U-test | **p=0.01** |
|  | duration | 33.875±3.989 | | 21.0±2.0 | | t-test | p=0.163 |
|  | onset | 4.125±0.915 | | 3.5±2.5 | | t-test | p=0.779 |
|  |  | NaCl  N=10 | | Yoh  N=10 | |  |  |
|  | frequency | 66.67%±0% | | 0%±0% | | Mann-Whitney U-test | **p=0.004** |
|  | duration | 27.0±6.792 | |  | |  |  |
|  | onset | 4.75±1.209 | | - | | - | - |
|  |  | NaCl  N=12 | | Clon  N=12 | |  |  |
|  | frequency | 83%±0% | | 0%±0% | | Mann-Whitney U-test | **p≤0.001** |
|  | duration | 44.6±4.799 | | - | | - | - |
|  | onset | 6.4±1.565 | | - | | - | - |
|  | | **mean±SEM** | | | | **Statistical test** | **p-value** |
|  | **parameter** | Ref | NE | | Yoh | paired t-test (or *Wilcoxon Signed Rank test) |  |
| **e** | SS/Sec | 46.514±6.083 | 5.8±3.245 | | 21.114±2.033 | *Ref vs NE | **p=0.016** |
|  |  |  |  |  |  | NE vs Yoh | **p=0.004** |
|  |  |  |  |  |  | Ref vs Yoh | **p=0.003** |
|  | predominant firing rate | 68.678±10.189 | 40.406±6.423 | | 44.332±7.817 | *Ref vs NE | **p=0.016** |
|  |  |  |  |  |  | NE vs Yoh | p=0.568 |
|  |  |  |  |  |  | Ref vs Yoh | **p≤0.001** |
|  | CV1 | 2.352±0.844 | 6.375±1.899 | | 3.607±0.910 | Ref vs NE | p=0.140 |
|  |  |  |  |  |  | NE vs Yoh | p=0.282 |
|  |  |  |  |  |  | Ref vs Yoh | p=0.124 |
|  | CV2 | 0.558±0.0787 | 0.821±0.118 | | 0.736±0.0534 | Ref vs NE | **p=0.029** |
|  |  |  |  |  |  | NE vs Yoh | p=0.405 |
|  |  |  |  |  |  | Ref vs Yoh | p=0.016 |
|  |  | **mean±SEM** | | | | **Statistical test** | **p-value** |
| **Figure 2** | **parameter** | Cacna1a^Citrine^  N=6 | | Cacna1a^purk(-/-)^  N=7 | |  |  |
| **b** |  | n=18 | | n=21 | |  |  |
|  | αDβH mFl [intensity/pixel] | 34.514±7.344 | | 63.171±4.198 | | Mann-Whitney U-test | **p≤0.001** |
|  | PC soma [µm^3^] | 285.697±13.991 | | 191.324±8.614 | | Mann-Whitney U-test | **p≤0.001** |
|  | ratio mFl/PC area | 0.125±0.00955 | | 0.340±0.0241 | | Mann-Whitney U-test | **p≤0.001** |
| **c** |  |  | |  | | Spermann Corellation |  |
| **d** | LC-DβH^+^ neurons | 509.167±20.78 | | 792.571±29.683 | | t-test | **p≤0.001** |
| **e** | LC surface [µm^2^] x 1000 | 62.639±6.288 | | 63.888±7.664 | | t-test | p=0.904 |
|  | DβH^+^ pixels/LC area | 57.110±2.363 | | 67.184±4.448 | | t-test | p=0.084 |
|  | DβH^+^ neurons/LC area | 0.00842±0.000605 | | 0.0134±0.00153 | | t-test | **p=0.016** |

Supplementary Table 1: Mean±SEM or % of mice, statistical tests and p-values for supplementary data.

| **Supplementary Figure 1** | **Statistical test** | **parameter** | **NaCl**  **N=10** | **Imiloxan**  **N=12** | | | | **p-value** |
| --- | --- | --- | --- | --- | --- | --- | --- | --- |
| a | One Way Rm ANOVA | frequency | % | mg/kg | | | % | p=0.835 |
|  |  |  | 58.33 | 0.1 | | | 58.33 |  |
|  |  |  |  | 0.3 | | | 58.33 |  |
|  |  |  |  | 1 | | | 50 |  |
|  |  |  |  | 3 | | | 58 |  |
|  |  |  |  | 10 | | | 75 |  |
|  |  |  | **NaCl** | **Imiloxan** | | | | **p-value** |
|  | One Way Rm ANOVA | duration | mean±SEM | mg/kg | | mean±SEM | | p=0.308 |
|  |  |  | 35.857±7.153 | 0.1 | | 33.571±3.598 | |  |
|  |  |  |  | 0.3 | | 52.286±12.537 | |  |
|  |  |  |  | 1 | | 52.333±9.387 | |  |
|  |  |  |  | 3 | | 51.429±9.921 | |  |
|  |  |  |  | 10 | | 39.75±5.897 | |  |
|  |  |  | **NaCl** | **Imiloxan** | | | | **p-value** |
|  | RM ANOVA on Ranks | onset | mean±SEM | mg/kg | mean±SEM | | | p=0.978 |
|  |  |  | 1.857±0.404 | 0.1 | 5.714±2.067 | | |  |
|  |  |  |  | 0.3 | 5.286±2.679 | | |  |
|  |  |  |  | 1 | 1.667±0.333 | | |  |
|  |  |  |  | 3 | 6.429±4.780 | | |  |
|  |  |  |  | 10 | 2.125±0.515 | | |  |

Supplementary Table 2: Yohimbine hydrochloride had no effect on motor performance in control Cacna1a^Citrine^ mice (n = 12).

| Test | Statistics | mean±SEM | | p-value | |  |
| --- | --- | --- | --- | --- | --- | --- |
|  |  | NaCl | Yohimbine | |  | |
| Pole test *time (s)* | Students T-tests | 43.458±6.840 | 35.458±9.692 | | p=0.088 | |
| Hang wire *time (s)* | Mann-Whitney Rank Sum Test | 57.0±1.639 | 49.389±13.56 | | p=0.241 | |
| Beam walk |  | | | | |  |
| *time (s)* | Students T-tests | 40.583±11.536 | 20.083±5.996 | | p=0.166 | |
| *idle (s)* | Students T-tests | 22.583±10.326 | 11.917±4.824 | | p=0.385 | |
| *falls (n)* | Students T-tests | 0±0 | 0±0 | | p=1 | |
| *right slips (n)* | Students T-tests | 0±0 | 0±0 | | p=1 | |
| *left slips (n* | Students T-tests | 0±0 | 0±0 | | p=1 | |
| Footprint Analysis |  | | | | |  |
| *length right front paw (cm)* | Students T-tests | 6.841±0.170 | 6.918±0.139 | | p=0.729 | |
| *length left front paw (cm)* | Students T-tests | 6.864±0.174 | 6.933±0.150 | | p=0.765 | |
| *length right hind paw (cm)* | Students T-tests | 6.780±0.156 | 7.304±0.209 | | p=0.564 | |
| *length left hind paw (cm)* | Students T-tests | 6.822±0.178 | 6.877±0.147 | | p=0.815 | |
| *width front paws (cm)* | Students T-tests | 1.404±0.0389 | 1.263±0.0316 | | **p=0.01** | |
| *width hind paws (cm)* | Students T-tests | 2.912±0.0926 | 2.598±0.0768 | | **p=0.016** | |
| Rotarod |  |  |  | |  | |
| *speed (rpm)* | Students T-tests | 18.889±0.520 | 16.250±0.991 | | **p=0.028** | |
| *time (min)* | Students T-tests | 127.542±9.858 | 145.111±6.755 | | p=0.156 | |
|  |  |  |  | |  | |
|  |  |  |  | |  | |
